# G-protein-coupled receptors mediate ω-3 PUFAs-inhibited colorectal cancer by activating the Hippo pathway

**DOI:** 10.18632/oncotarget.11089

**Published:** 2016-08-05

**Authors:** Kun Zhang, Zhimei Hu, Haixia Qi, Zhemin Shi, Yanan Chang, Qingbin Yao, Hongmei Cui, Lina Zheng, Yawei Han, Xiaohui Han, Zhen Zhang, Ting Chen, Wei Hong

**Affiliations:** ^1^ Department of Histology and Embryology, School of Basic Medical Sciences, Tianjin Medical University, Tianjin 300070, China

**Keywords:** omega-3 PUFAs, Hippo pathway, GPR, colorectal cancer, YAP

## Abstract

Colorectal cancer (CRC) is one of the most common cancers leading to high mortality. However, long-term administration of anti-tumor therapy for CRC is not feasible due to the side effects. Omega-3 polyunsaturated fatty acids (ω-3 PUFAs), particularly DHA and EPA, exert protection against CRC, but the mechanisms are unclear. Here, we show that ω-3 PUFAs inhibit proliferation and induce apoptosis of CRC cells *in vitro* and alleviate AOM/DSS-induced mice colorectal cancer *in vivo*. Moreover, ω-3 PUFAs promote phosphorylation and cytoplasmic retention of YAP and this effect was mediated by MST1/2 and LATS1, suggesting that the canonical Hippo Pathway is involved in ω-3 PUFAs function. We further confirmed that increase of pYAP by ω-3 PUFAs was mediated by GPRs, including GPR40 and GPR120, which subsequently activate PKA via Gαs, thus inducing the Hippo pathway activation. These data provide a novel DHA/EPA-GPR40/120-Gαs-PKA-MST1/2-LATS1-YAP signaling pathway which is linked to ω-3 PUFAs-induced inhibition of cell proliferation and promotion of apoptosis in CRC cells, indicating a mechanism that could explain the anti-cancer action of ω-3 PUFAs.

## INTRODUCTION

Colorectal cancer (CRC) remains a significant cause of morbidity and mortality worldwide. The global burden of CRC is expected to increase by 60% to more than 2.2 million new cases and 1.1 million deaths by the year 2030 [1, 2]. Dietary omega-3 polyunsaturated fatty acids (ω-3 PUFAs), particularly eicosapentaenoic acid (EPA, 20:5) and docosahexaenoic acid (DHA, 22:6), have been reported to be generally beneficial in the onset and progression of several chronic diseases, including coronary artery disease, atherosclerosis, diabetes and cancers [3–6]. Particularly, many clinical and experimental data clearly support the idea that consumption of diets rich in ω-3 PUFAs not only slows the growth of colorectal cancer but also increases sensitivity to chemotherapy [7–11]. Nevertheless, despite intense investigation, the precise molecular mechanisms responsible for the anti-tumor properties of ω-3 PUFAs remain elusive.

In metazoan, the size of each organ is elaborately modulated by the coordination between cell proliferation and cell death. Many signaling pathways are involved in this process, and their dysregulation can lead to uncontrolled growth and cancinogenesis. A newly established tumor suppressor pathway-Hippo pathway has gained more and more attention [12]. Mechanically, MST1/2 (Hpo in Drosophila) serves as upstream kinases associated with its scaffolding partner SAV1 (Salvador in Drosophila) and phosphorylates LATS1/2 (Warts in Drosophila) and MOB1 (Mats in Drosophila). Activated LATS1/2 kinases then phosphorylate YAP/TAZ (Yki in Drosophila), leading to the inactivation of YAP/TAZ by sequestering in the cytoplasm via interaction with 14-3-3 proteins or proteasome-mediated degradation (Supplementary Figure 1A). In the nucleus, YAP/TAZ can associate with a number of transcriptional factors to mediate the transcription of genes that control cell proliferation and apoptosis [13–18]. In addition, many studies have been devoted to identify upstream regulators of the Hippo pathway in order to elucidate the mechanisms underlying organ size control. G-protein-coupled receptors (GPRs), the largest family of cell surface receptors, have also been reported to function upstream of the Hippo pathway through Rho GTPase and cytoskeleton remodeling [19–21].

Notably, inactivation of the Hippo pathway components and activation of YAP/TAZ have been observed in various cancers including colorectal cancer [22–24]. Decreased *Mst1/2* expression has been observed in human colorectal cancers [25]. Most significantly, YAP/TAZ expression is elevated in human colorectal cancers compared with its expression in para-tumor tissue and YAP/TAZ expression may be used as a CRC prognostic marker [23, 24]. Moreover, YAP can promote resistance of CRC cells to chemotherapy. Mechanically, the story behind YAP/TAZ is rather complex as the Hippo pathway cross talks with multiple signaling pathways [26–28] involved in CRC, and some of these pathways, such as the Wnt pathway [26, 28–32], is aberrantly activated and mutations in components of the Wnt pathway, most often in APC, are found in 90% of colon cancers. As such, members of the Hippo pathway are emerging targets in anti-cancer treatments.

In the present study, we showed that ω-3 PUFAs inhibit proliferation and induce apoptosis of CRC cells *in vitro* and alleviate AOM/DSS (azoxymethane/dextran sulfate sodium)-induced colorectal cancer *in vivo*. Mechanically, a novel DHA/EPA-GPR40/120-Gαs-PKA-MST1/2-LATS1-YAP signaling pathway was linked to ω-3 PUFAs-induced inhibition of cell proliferation and promotion of apoptosis in CRC cells. Our findings suggest a novel mechanism that could explain the anti-cancer action of ω-3 PUFAs.

## RESULTS

### Intake of ω-3 PUFAs prevents AOM/DSS-induced colorectal cancer

**Figure 1:**
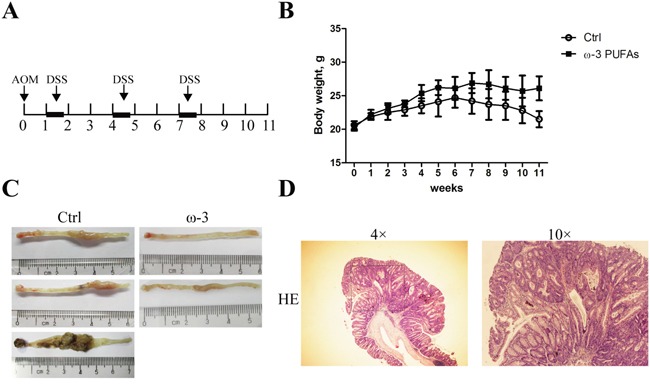
ω-3 PUFAs intake prevents AOM/DSS-induced colorectal cancer **A.** Tumor induction protocol. Mice were injected with AOM on day 1 followed by three rounds of DSS treatment. After 1 week, 2.5% DSS was administered in the drinking water for 5 days, followed by 16 days of tap water in sterile. This cycle was repeated twice (5 days of 2.5% DSS followed by a 16 days recovery period and 5 days of 2.5% DSS). Mice were terminated at week 11 after injection of AOM. **B.** Body weight changes in mice fed with different diets. Values are means ± SEM. **C.** Photographs showing colons with tumors from mice fed with the control diet (left) and mice fed with the diet supplemented with ω-3 PUFAs (right). **D.** Representative hematoxylin and eosin stain of colorectal tumor.

During the induction of CRC, mice were fed a diet supplemented with ω-3 PUFAs for 11 weeks (Figure 1A). This decreased the ω-6 PUFAs (18:2 n-6, 20:4 n-6, and 22:4 n-6) content and increased the ω-3 PUFAs (20:5 n-3, 22:5 n-3, and 22:6 n-3) content in both serum and colonic mucosa relative to mice on the control diet (Supplementary Table 1). Treatment with AOM/DSS resulted in a tumor incidence of 55% in mice fed the diet supplemented with ω-3 PUFAs as compared with 93.3% in mice fed the control diet. Moreover, there were significantly fewer tumors in the ω-3 group mice than in control group. In addition, tumors in ω-3 group mice on average tended to be smaller compared to control group mice (Table 1). Interestingly, the ω-3 group mice were heavier than those in control group since the week 4 (Figure 1B). Taken together, our data indicated that dietary supplementation with ω-3 PUFAs protects against AOM/DSS-induced colorectal cancer.

**Table 1:** Colorectal tumor induction in control and ω-3 PUFAs group mice

	Control	ω-3 PUFAs
Tumor incidence	93.3% (14/15)	55%(11/20)^*^
Average number of tumors	4.17±1.56	1.83±0.56^*^
Tumor volume (mm^3^)	25.75±13.84	12.40±5.36^*^

Values are means ± SEM.

* indicate statistically significant differences with *P*<0.05, compared to the control group.

### ω-3 PUFAs suppress proliferation and induce apoptosis of CRC cells

**Figure 2:**
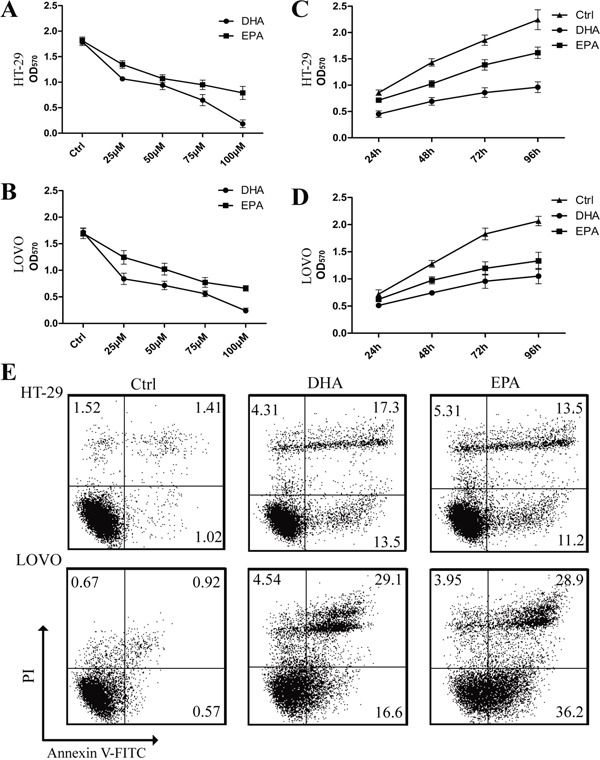
ω-3 PUFAs suppress proliferation and induce apoptosis of CRC cells **A-D**. HT-29 (A) and LOVO (B) cells were assessed by an MTT assay for viability following exposure for 72 h to media containing 10% FBS and varying concentrations of DHA or EPA. HT-29 (C) and LOVO (D) cells were assessed by an MTT assay for viability following exposure for 24h, 48h, 72h and 96h, respectively, to media containing 10% FBS and 75μM DHA or EPA. **E.** HT-29 and LOVO cells were treated with 75 μM DHA or EPA for 48 h, cell apoptosis was determined by FACS analysis. The data are expressed as the mean ± SEM for triplicate experiments. *P<0.05.

To explain the anti-tumor effect of dietary ω-3 PUFAs observed *in vivo*, we investigated the effect of DHA and EPA on the proliferation and apoptosis of HT-29 and LOVO cells by MTT and flow cytometry assays. Firstly, we used increasing amounts up to 100μM DHA or EPA to treat the CRC cells for 6 h, respectively, to evaluate whether ω-3 PUFAs has a toxic effect. Trypan blue staining showed no difference in the cells treated with either DHA or EPA compared with the control cells (Supplementary Figure 1B and 1C), suggesting that the concentrations of ω-3 PUFAs used in the present study caused no toxic effect on CRC cells. Furthermore, 75μM of DHA or EPA significantly increased the ω-3 PUFAs content in CRC cells (data not shown). The results of MTT showed that ω-3 PUFAs inhibit the proliferation of CRC cells and the inhibitory effect was enhanced with increased amount of ω-3 PUFAs, with the maximal effect at 100μM, indicating that the inhibitory effect of ω-3 PUFAs on CRC cells growth is dose-dependent (Figure 2A and 2B). Meanwhile, when CRC cells were treated with 75μM ω-3 PUFAs for increased time, the inhibitory effect of ω-3 PUFAs on the cell growth exhibits a time-dependent manner (Figure 2C and 2D). In addition, FACS with Annexin V and PI double staining was applied for apoptosis analysis. When CRC cells were treated with 75μM ω-3 PUFAs for 24h and 48h, there were more apoptotic and fewer survival populations (Figure 2E and Supplementary Figure 1D). Taken together, these results indicate that ω-3 PUFAs suppress CRC cells proliferation and induce apoptosis *in vitro*.

### ω-3 PUFAs induce phosphorylation and cytoplasmic translocation of YAP in CRC cells

**Figure 3:**
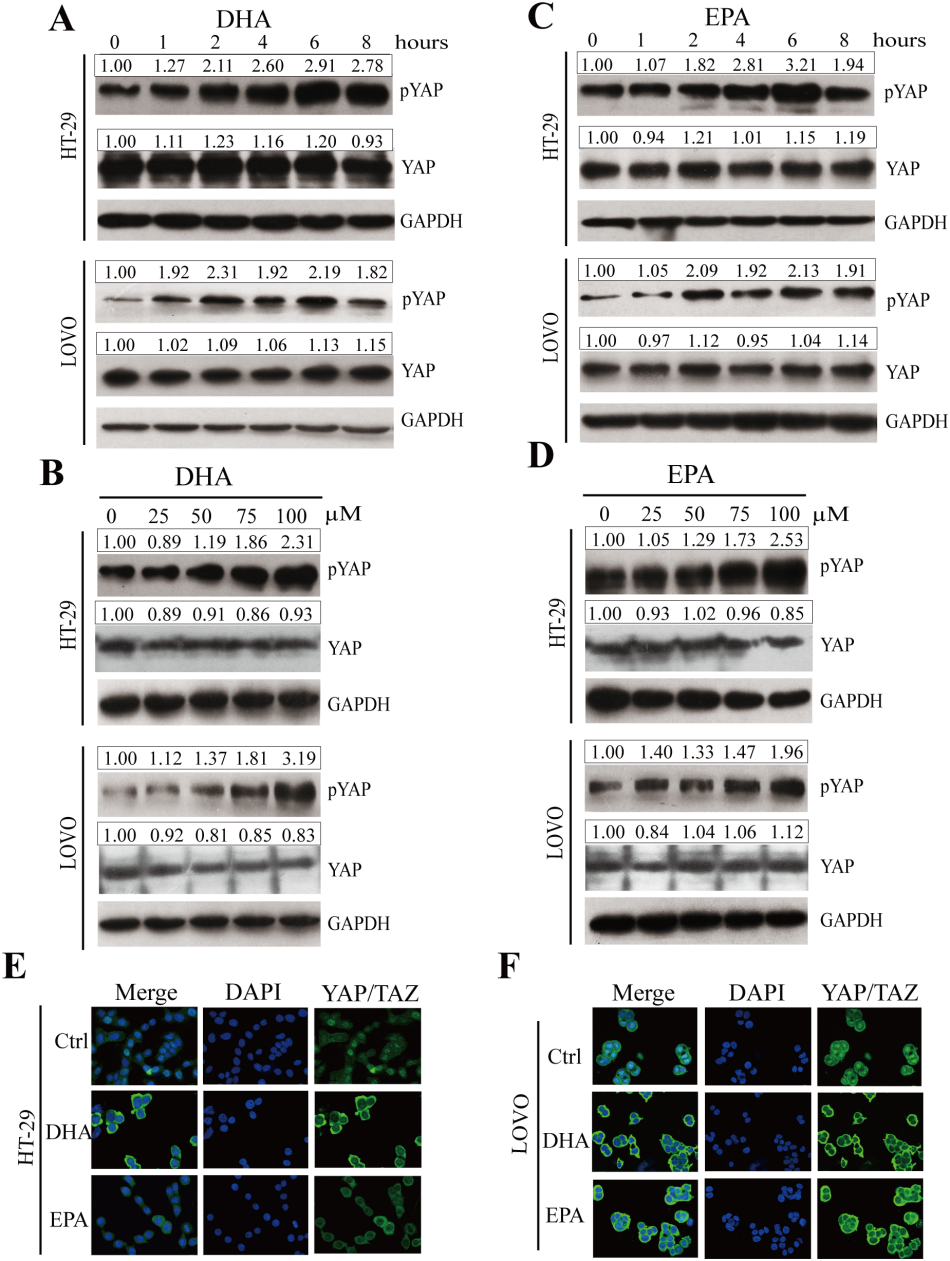
ω-3 PUFAs induce phosphorylation and cytoplasmic translocation of YAP in CRC cells **A-D.** pYAP and YAP signals were examined and quantified by western blot after HT-29 and LOVO cells were treated with 75 μM DHA (A and B) or EPA (C and D) for indicated times (A and C) or different concentrations at 4h (B and D). GAPDH served as the loading control. Bands were semiquantified by image intensity area under the curve. Intensity of specific band is normalized in relation to loading control protein intensity. **E** and **F.** HT-29 (E) and LOVO (F) cells were treated with 75 μM DHA or EPA for 6 h. Expression and translocation of YAP/TAZ were determined by confocal microscopy; DAPI stained nuclei blue.

The Hippo pathway has been proposed to restrict organ size, and dysregulation of this pathway leads to tumorigenesis [13, 22–24, 33]. To investigate whether the Hippo-YAP pathway is correlated with the effect of ω-3 PUFAs on CRC cells, we firstly tested whether the phosphorylation of YAP is promoted by ω-3 PUFAs. HT-29 and LOVO cells were treated with DHA or EPA for indicated times and lysed for immunoblot. The result showed that DHA or EPA promotes YAP phosphorylation (pYAP, S127) and the increase of pYAP caused by ω-3 PUFAs displayed a time-dependency, while the protein level of total YAP is unchanged (Figure 3A and 3C). In addition, DHA or EPA promotes YAP phosphorylation also in a dose-dependent manner (Figure 3B and 3D). It has also been reported that YAP is preferentially localized to nuclei in cells, while the Hippo signaling pathway antagonizes YAP function by promoting its cytoplasmic localization in an S127 phosphorylation-dependent manner. In this study, we performed confocal experiment with HT-29 (Figure 3E) and LOVO (Figure 3F) cells treated by DHA or EPA to investigate whether ω-3 PUFAs influence the translocation of YAP. The result revealed that YAP/TAZ shifted from the nucleus to the cytoplasm when the cells were treated by DHA or EPA, consistent with the finding, by western blot analyses, that ω-3 PUFAs promote YAP phosphorylation. Taken together, these data demonstrate that ω-3 PUFAs induce YAP phosphorylation and cytoplasmic retention in CRC cells.

### ω-3 PUFAs inhibit proliferation and induce apoptosis of CRC cells via YAP

**Figure 4:**
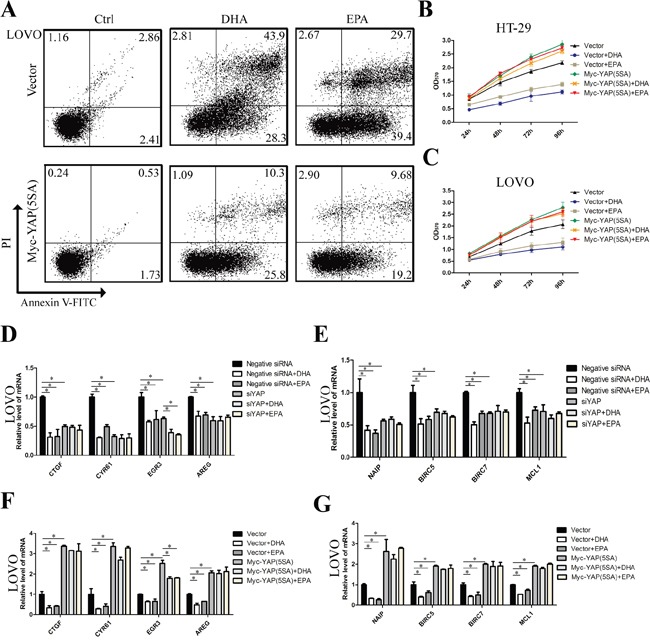
ω-3 PUFAs inhibit proliferation and induce apoptosis of CRC cells via YAP **A.** LOVO cells were infected with empty vector or pQCXIH Myc-YAP (5SA) retroviral for 72 h, after infection, cells were treated with 75 μM DHA or EPA for additional 48 h, cell apoptosis was determined by FACS analysis. **B** and **C.** CRC cells were infected with empty vector or pQCXIH Myc-YAP (5SA) retroviral for 72 h, after infection, cells were treated with 75 μM DHA or EPA for 24h, 48h, 72h, or 96h respectively, cell viability was determined by MTT assay. **D** and **E.** LOVO cells were transfected with YAP siRNA for 48 h. After transfection, cells were treated with 75μM DHA or EPA for additional 24h. Total RNA was extracted and used for qRT-PCR analysis of the representative panel of pro-proliferative genes and anti-apoptosis genes. **F** and **G.** LOVO cells were infected with empty vector or pQCXIH Myc-YAP (5SA) retroviral for 72 h. After infection, cells were treated with 75 μM DHA or EPA for additional 24 h. Total RNA was extracted and used for qRT-PCR analysis of the representative panel of pro-proliferative genes and anti-apoptosis genes. The data are expressed as the mean ± SEM for triplicate experiments. *P<0.05.

To determine whether ω-3 PUFAs inhibit proliferation and induce apoptosis of CRC cells are mediated by YAP, which directly promotes the expression of many proliferation and apoptosis related genes, including CTGF, AREG, EGR3, Cyr61, the IAP (inhibitor of apoptosis proteins) family members NAIP, BIRC5 and BIRC7, and the BCL2 family gene MCL1 [12, 18, 21], we infected the CRC cells by YAP (5SA), a constitutively active YAP mutant, to over-express YAP. The infected cells were then treated by ω-3 PUFAs and were used for flow cytometry and MTT assays to test the apoptosis and proliferation, respectively. The results showed that there were fewer apoptotic and more survival populations in YAP (5SA)-infected cells than in control cells (Figure 4A and Supplementary Figure 2A), suggesting that YAP over-expression results in reduced apoptosis induced by ω-3 PUFAs. In addition, the result of MTT assay revealed that over-expression of YAP (5SA) caused an increased proliferation than control cells, and subsequent treatment of ω-3 PUFAs could not further inhibit the CRC cells proliferation (Figure 4B and 4C). Moreover, the CRC cells were transfected with YAP specific siRNA which could effectively knock down the mRNA and the subsequent protein levels of YAP (Supplementary Figure 3A-3D). Additionally, the mRNA levels of *CTGF, Cyr61, EGR3, AREG, NAIP, BIRC5, BIRC7* and *MCL1*, in CRC cells treated by ω-3 PUFAs were dramatically reduced as shown by qRT-PCR analysis. Furthermore, knockdown of YAP decreases, while over-expression of YAP increases the expression of these targeted genes in CRC cells and abolished ω-3 PUFAs-induced inhibitory effect (Figure 4D-4G and Supplementary Figure 2B-2E). One exception is EGR3, which still presents a slight decline when the cells were treated by ω-3 PUFAs. The possible reason for this could be other signaling pathways are involved in ω-3 PUFAs-decreased EGR3 expression apart from YAP. Taken together, our data revealed that ω-3 PUFAs inhibit proliferation and induce apoptosis of CRC cells through YAP.

### ω-3 PUFAs-induced YAP phosphorylation and cytoplasm translocation is predominantly through the canonical Hippo pathway

**Figure 5:**
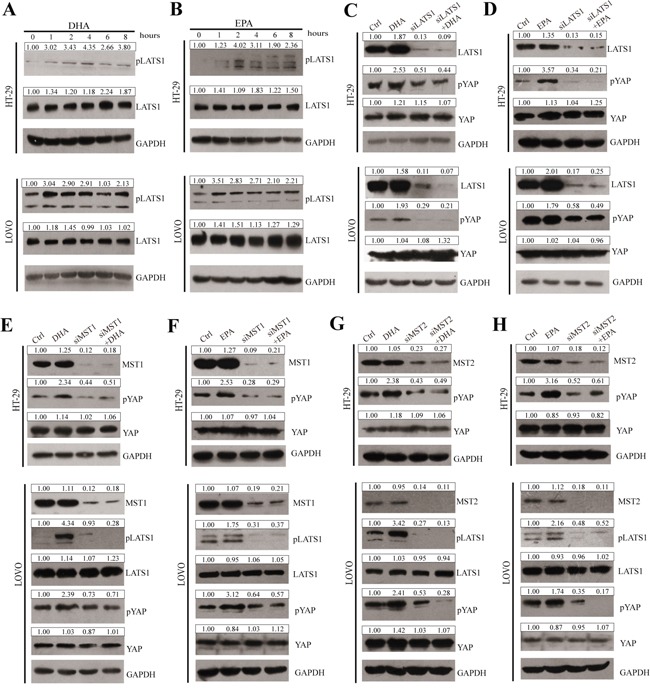
ω-3 PUFAs-induced YAP phosphorylation and cytoplasm translocation is predominantly through the canonical Hippo Pathway **A** and **B.** pLATS1 and LATS1 expressions were examined and quantified by western blot after HT-29 and LOVO cells were treated with 75μM DHA (A) or EPA (B) for indicated times. **C** and **D.** CRC cells were transfected with LATS1 siRNA for 48h, after transfection, cells were treated with 75μM DHA (C) or EPA (D) for additional 4h, pYAP, YAP and LATS1 expressions were examined and quantified by western blot in HT-29 cells and LOVO cells. **E** and **F.** CRC cells were transfected with MST1 siRNA for 48h, after transfection, cells were treated with 75μM DHA (E) or EPA (F) for additional 4h. pYAP, YAP, pLATS1, LATS1 and MST1 expressions were examined and quantified by western blot in HT-29 cells and LOVO cells. **G** and **H.** CRC cells were transfected with MST2 siRNA for 48 h. After transfection, cells were treated with 75 μM DHA (G) or EPA (H) for additional 4 h. pYAP, YAP, pLATS1, LATS1 and MST2 expressions were examined and quantified by western blot in HT-29 cells and LOVO cells. GAPDH served as the loading control. Bands were semiquantified by image intensity area under the curve. Intensity of specific band is normalized in relation to loading control protein intensity.

The kinase cascade of MST1/2 and LATS1/2 represents a core component of the mammalian Hippo pathway [12, 14, 16, 17]. We intend to explore the possibility that ω-3 PUFAs phosphorylate YAP through the canonical Hippo pathway. As shown in Figure 5A and 5B, phosphorylation of LATS1 protein remarkably increased in 75μM ω-3 PUFAs-treated HT-29 and LOVO cells, and the increasing peaks of pLATS1 are at 4h and 2h, respectively, which are earlier than those of pYAP (Figure 3A and 3C). Interestingly, the protein levels of total LATS1 are also slightly up-regulated by ω-3 PUFAs. As the expression of total LATS2 cannot be detected in CRC cells, LATS1 was therefore knocked down in HT-29 and LOVO cells by specific siRNA. The results showed that knockdown of LATS1 dramatically reduced basal pYAP levels and abolished ω-3 PUFAs-increased pYAP levels (Figure 5C and 5D). Furthermore, knockdown of MST1 or MST2 by specific siRNA also reduced basal pLATS1 and pYAP levels, and subsequent treatment of ω-3 PUFAs could not further increase pLATS1 and pYAP due to knockdown of MST1 or MST2 (Figure 5E-5H). These results suggest phosphorylation of YAP and its cytoplasmic retention caused by ω-3 PUFAs were through the canonical Hippo pathway.

### GPR120, GPR40, Gαs and PKA are involved in mediating ω-3 PUFAs-induced YAP phosphorylation

**Figure 6:**
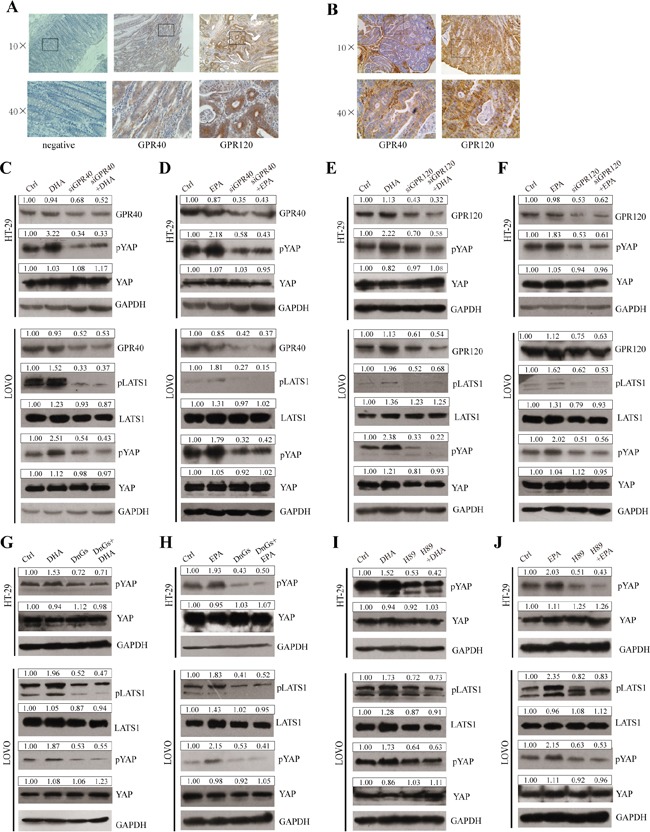
GPR120, GPR40, Gαs and PKA are involved in mediating ω-3 PUFAs-induced YAP phosphorylation **A** and **B.** The expressions of GPR40 and GPR120 in paraffin-embedded CRC tissues of human CRC patients (A) and AOM/DSS-induced mouse modles (B) were examined using IHC staining, 10× and 40×magnification. **C** and **D.** GPR40 was knocked down by siRNA and further treated with 75μM DHA (C) or EPA (D) for additional 4h. The expression of pLATS1, LATS1, pYAP, YAP and GPR40 were examined by western blot and quantified in HT-29 and LOVO cells. **E** and **F.** GPR120 was knocked down by siRNA and further treated with 75μM DHA (E) or EPA (F) for additional 4h. The expression of pLATS1, LATS1, pYAP, YAP and GPR120 were examined by western blot and quantified in HT-29 and LOVO cells. **G** and **H.** Gαs function was blocked by transfected with DnGs and further treated with 75μM DHA (G) or EPA (H) for additional 4h. The expression of pLATS1, LATS1, pYAP and YAP were examined by western blot and quantified in HT-29 and LOVO cells. **I** and **J.** PKA was inhibited by the inhibitor H-89 and further treated with 75μM DHA (I) or EPA (J) for additional 4h. The expression of pLATS1, LATS1, pYAP and YAP were examined by western blot and quantified in HT-29 and LOVO cells. GAPDH served as the loading control. Bands were semiquantified by image intensity area under the curve. Intensity of specific band is normalized in relation to loading control protein intensity.

Free fatty acids (FFAs) can act as ligands of several GPRs [6, 34–38]. In addition, it has been reported that GPRs function upstream of the Hippo pathway through Rho GTPase and cytoskeleton remodeling [21]. Therefore, we intend to investigate whether ω-3 PUFAs trigger the canonical Hippo pathway via the GPRs and theirs downstream actors. Firstly, we examined the expression of GPR40 and GPR120 in paraffin-embedded CRC tissues of patients using IHC staining. As shown in Figure 6A and 6B, both of the two GPRs exist in cancerous tissues both from the human CRC patients and the AOM/DSS-induced CRC mouse model, inconsistent with the previous study which demonstrated that GPR40 could not be detected in CRC tissues [39]. Secondly, to determine whether GPR40 and GPR120 inhibit YAP activation, we performed loss of function of GPR40 and GPR120 on YAP phosphorylation in CRC cells. Our data indicate that knockdown of GPR40 or GPR120 through siRNA could significantly block ω-3 PUFAs-induced increase of LATS1 and YAP phosphorylation, suggesting that ω-3 PUFAs trigger the Hippo pathway via GPR40 and GPR120 (Figure 6C-6F). We then inhibited G protein Gαs activity by using the dominant-negative Gαs mutant (DnGs) to investigate whether ω-3 PUFAs-induced activation of the Hippo pathway via Gαs, a downstream effector of GPR40 and GPR120. The result from CRC cells transfected with DnGs showed the levels of pLATS1 and pYAP were decreased, even though further treatment of ω-3 PUFAs could not reverse this decreasing tendency, suggesting that Gαs protein is essential for ω-3 PUFAs-induced YAP phosphorylation (Figure 6G and 6H). Since it has been reported that cAMP-dependent protein kinase A (PKA) which can be activated by Gαs could promote LATS-induced YAP phosphorylation [40, 41], we investigate whether PKA is involved in ω-3 PUFAs-induced YAP phosphorylation. Therefore, we pretreated CRC cells with H-89, a PKA inhibitor, and immunoblot analysis with these cells demonstrated that the levels of both pLATS1 and pYAP greatly reduced by H-89 treatment. However, further treatment with ω-3 PUFAs could not increase the phosphorylation of both proteins (Figure 6I and 6J). Taken together, our results revealed that ω-3 PUFAs promote YAP phosphorylation via GPR40/120-Gαs-PKA cascade in CRC cells.

### ω-3 PUFAs supplementation prevents AOM/DSS-induced colorectal cancer through activating the Hippo pathway

**Figure 7:**
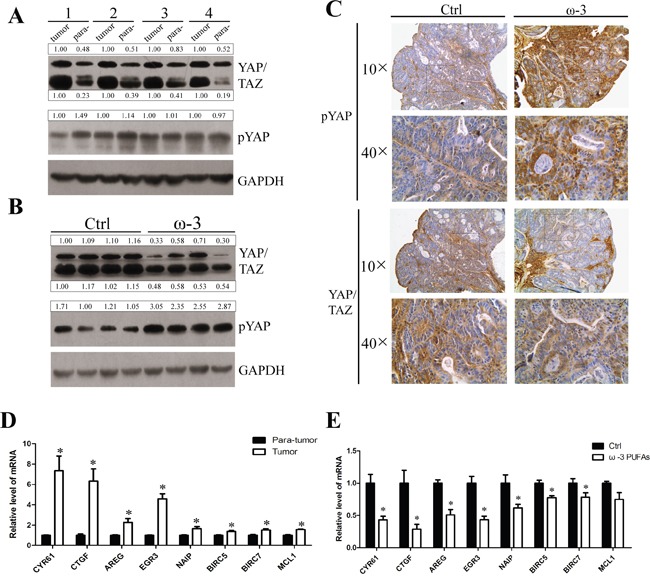
ω-3 PUFAs supplementation prevents AOM/DSS-induced colorectal cancer through activating the Hippo pathway **A** and **B.** Total protein of the colorectal tumor tissues and para-tumor normal tissues from the control group mice (A) or total protein of colorectal tumor tissues from the control group mice and ω-3 group mice (B) were prepared and subjected to western blot analysis to detect YAP/TAZ and pYAP. GAPDH served as the loading control. Bands were semiquantified by image intensity area under the curve. Intensity of specific band is normalized in relation to loading control protein intensity. **C.** IHC stainings of YAP/TAZ and pYAP in colorectal tumors from the control group mice (left panel) and the ω-3 group mice (right panel), 10× and 40×magnification. **D** and **E.** Total RNA of the colorectal tumor tissues and para-tumor normal tissues from the control group mice (D), or total RNA of colorectal tumor tissues from the control group and ω-3 group mice (E) was prepared and used for qRT-PCR analysis of pro-proliferative genes and anti-apoptosis genes. The data are expressed as the mean ± SEM for triplicate experiments. *P<0.05.

To confirm the anti-tumor mechanism of ω-3 PUFAs observed *in vitro*, we further investigated whether ω-3 PUFAs supplementation prevents AOM/DSS-induced colorectal cancer through activating the Hippo pathway. Firstly, western blotting was applied to determine the proteins of YAP and TAZ in CRC tissues, and the results demonstrated that YAP and TAZ proteins increased in CRC tissues compared with normal tissues. However, the expression of YAP and TAZ dramatically decreased in carcinomatous tissues derived from ω-3 treatment group versus control group (Figure 7A and 7B). In addition, pYAP was significantly increased in tumor tissues of ω-3 PUFAs group, compared with control group (Figure 7A and 7B), consistent with the data obtained from CRC cells. Moreover, IHC experiments detecting YAP and pYAP in colorectal tumor sections also showed that pYAP was increased, but YAP/TAZ were decreased in ω-3 PUFAs group compared with control (Figure 7C). Finally, qRT-PCR analysis of mRNAs isolated from colorectal tumor and normal tissues revealed that the YAP targeted genes related to proliferation and apoptosis were increased in CRC tissues compared with normal tissues, and the increase of these genes diminished distinctly after ω-3 PUFAs intake (Figure 7D and 7E). Taken together, ω-3 PUFAs could inhibit CRC occurrence and development in AOM/DSS models via the Hippo-YAP pathway.

## DISCUSSION

**Figure 8:**
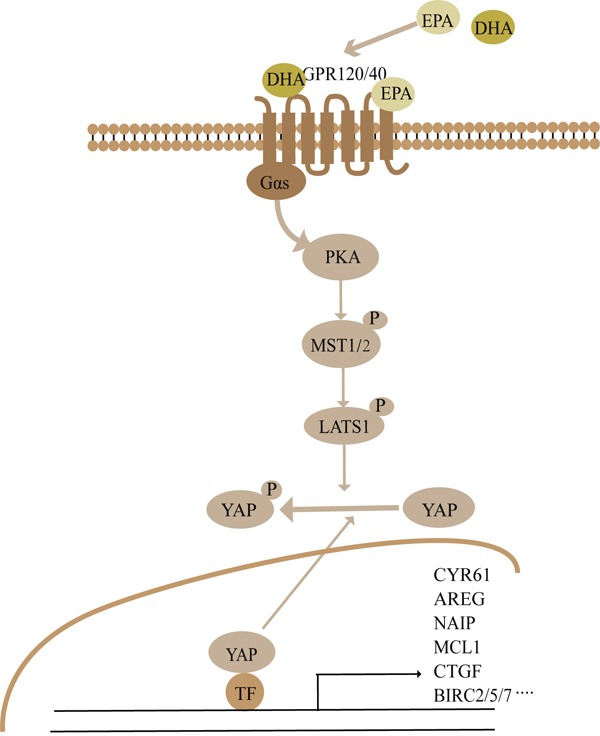
Summary the ω-3 PUFAs-YAP signaling pathway revealed in this study See Discussion for details.

In the present study, we investigated the effects of ω-3 PUFAs on the colorectal cancer. Even though a variety of experimental studies and different clinical trials have shown a reduced incidence of colorectal cancer in populations consuming high levels of fish [7, 9, 10, 42]. However, the precise molecular mechanisms responsible for the anti-tumor properties of ω-3 PUFAs have not been identified. Here, using HT-29 and LOVO colorectal cancer cells and AOM/DSS-induced colorectal cancer model, we provided convincing evidence that ω-3 PUFAs could efficiently prevent colorectal carcinogenesis and tumor development by inhibition of CRC cell proliferation and induction of apoptosis through activating the Hippo pathway. In addition, our results revealed that ω-3 PUFAs promote the Hippo pathway activation, phosphorylation and cytoplasmic retention of YAP via GPRs (GPR40 and GPR120)-Gαs-PKA cascade (Figure 8).

A number of researches revealed that the pathway upstream of YAP phosphorylation is operative in a tissue- or context-specific manner, which suggests the existence of non-canonical components of the Hippo kinase cascade. Yu et al. [21] have shown that LPA acivates YAP mainly via suppression of LATS1/2 other than MST in HEK293A or MEF cells. While in OVCA433 cells, LPA induces YAP dephosphorylation not by LATS1/2 [43]. We now demonstrate a distinct signaling pathway that ω-3 PUFAs are benefit in prevention of CRC by activating the canonical Hippo-YAP pathway. Firstly, ω-3 PUFAs could successively phosphorylate LATS1 and YAP, the two central members of the Hippo signaling both in CRC cell lines and AOM/DSS-induced CRC tissues. Moreover, the increasing peak of pLATS1 is earlier than that of pYAP, suggesting that ω-3 PUFAs promote YAP phosphorylation through LATS1. Secondly, stimulation with ω-3 PUFAs could no longer induce pYAP increase by knockdown of MST1, MST2 or LATS1. This indicated the core of the Hippo pathway, MST1/2 or LATS1, are involved in the induction of ω-3 PUFAs on YAP phosphorylation. However, the expression of pMST1/2 and LATS2 cannot be detected in CRC cells by western blot. Taken together; our data reveal that ω-3 PUFAs activate YAP through the canonical Hippo pathway.

There are a large quantity of important studies showing direct interactions between PUFAs and GPRs [34, 36–38]. GPR120 functions as an ω-3 PUFAs receptor/sensor in pro-inflammatory macrophages and mature adipocytes, while GPR40 functions as a specific receptor for long-chain FFAs and plays a role in pancreatic β-cell, neurological function and the growth of human melanoma. In addition, GPRs have been reported to function upstream of the Hippo pathway through Rho GTPase and cytoskeleton remodeling [19, 21, 44]. We assume that ω-3 PUFAs activate GPR members, subsequently phosphorylate YAP through a series of kinases cascade. Strikingly, we detected that GPR40 presents comparable expression as GPR120 in CRC tissues from patients, AOM/DSS-induced mice and colonic cancer cell lines, which is incompatible with a previous report [39]. The discrepancy may caused by different anti-GPR40 antibody they used in IHC, and immunoblot analysis to detect GPR40 was not performed in that study. Moreover, loss of GPR40 or GPR120 could largely block ω-3 PUFAs-induced increase of LATS1 and YAP phosphorylation. Therefore, the activity of LATS1 kinase and YAP are robustly regulated by GPRs. Subsequently, we investigated the actors involved in the signaling from GPRs to Hippo-YAP. Gαs is a stimulatory subunit of G protein and it plays an essential role in GPR-mediated signal transduction by coupling the receptors to the activation of AC and increases the synthesis of cAMP [21]. The accumulation of cAMP could turn on effector kinases, including PKA. In addition, Yu et al. [21] demonstrated GPRs that mainly activate Gs signaling could induce YAP/TAZ phosphorylation, while cAMP and PKA may function as a bridge between Gs-protein signals and Hippo pathway. In the present study, we found ω-3 PUFAs promote GPRs, GPR40 and GPR120, activation which has been linked to Gαs and PKA, ultimately activate the Hippo pathway.

In conclusion, we discovered an anti-tumor effect of ω-3 PUFAs and established for the first time that the Hippo-YAP pathway is the crucial signaling which mediates ω-3 PUFAs-induced CRC inhibition. Mechanically, a novel DHA/EPA-GPR40/120-Gαs-PKA-MST1/2-LATS1-YAP signaling pathway was linked to ω-3 PUFAs-induced inhibition of cell proliferation and promotion of apoptosis in CRC cells (Figure 8). Overall, our findings suggest a novel mechanism that could explain the anti-cancer action of ω-3 PUFAs.

## MATERIALS AND METHODS

### Cell lines and fatty acid treatment

HT-29 and LOVO cells were obtained from the Shanghai Institute of Cell Biology, Chinese Academy of Sciences and maintained in DMEM/F12 medium (Gibco/BRL, Gaithersburg, MD) supplemented with 10% FBS (Gibco, Gaithersburg, MD), 100U/ml of penicillin and 100μg/ml of streptomycin at 37°C and in an atmosphere of 5% CO_2_. Stock solutions of DHA and EPA in ethanol were stored at −20°C.

### Animals and tumor induction

All Balb/c mice aged at 8 weeks obtained from Institute of Laboratory Animal Sciences, CAMS and PUMC (Beijing, China). Mice were maintained in a 12-h light/dark cycle at 22- 25°C with free access to food and water. All protocols were approved by the Ethical Committee of Tianjin Medical University. After acclimatization for one week, mice were assigned at random to 2 groups. Control group (n=15) was fed with AIN 93 diet during the period of experiment, while the experimental group (n=20) was fed on a modified AIN 93 diet containing 10% (wt/wt) of a fish oil concentrate containing 33% EPA and 23% DHA during the period of experiment. Treatment scheme is summarized in Figure 1A. For tumor induction, both groups were injected intraperitoneally with the genotoxic carcinogen AOM (10 mg/kg, Sigma-Aldrich) followed by three rounds of DSS treatment. After 1 week, 2.5% DSS (molecular weight, 36,000-50,000; MP Biomedicals) was administered in the drinking water for 5 days, followed by 16 days of tap water in sterile. This cycle was repeated twice (5 days of 2.5% DSS followed by a 16 days recovery period and 5 days of 2.5% DSS). Mice were terminated at 11 week after injection of AOM. Subsequently, each colon was resected proximally at the junction between the cecum and distally at the anus, flushed with PBS. Gross examination was performed to evaluate the pattern of tumor development, including quantity and size of each tumor within the large bowel. In addition, the incidence (defined as number of mice with tumors/total mice in the group), the mean number of tumors/mouse ± standard deviation, as well as the mean tumor volume in the group ± standard deviation was calculated for each group. For tumor volume, we used a common approximated formula: V=0.5× length×width^2^.

### Measurement of fatty acids

Fatty acids in serum and colon mucosa were measured using the method of Kang et al [45, 46]. A similar analysis was performed on CRC cells. Briefly, C17:0 (1 mg/ml in hexane) (Sigma, St. Louis, MO) was added to each sample as an internal standard and the total lipids extracts from plasma and colon mucosa from each feeding group were based on the methods of Folch [47]. Fatty acid methyl esters were prepared by heating at 90–110°C for 1 h under BF3/methanol reagent (14% Boron Trifluoride). Fatty acid methyl esters were analyzed by gas chromatography using a fully automated HP5890 system equipped with a flame ionization detector. The chromatography utilized an Omegawax 250 capillary column (30m×0.25mm I.D.). Peaks were identified by comparison of retention times with external FA methyl ester standard mixtures from NuCheck Prep (Elysian, MN). Results were normalized for weight percent of each FA.

### Cell proliferation assay

Cell growth was assessed by MTT [3,(4, 5-dimethylthiazol-2-yl) 2, 5-diphenyltetrazoliumbromide] (Sigma, St. Louis, MO) dye conversion at 570 nm following manufacturer's instructions. Briefly, Cells (8×10^3^/well) were plated in 96-well plates and allowed to attach for 24h, and then cultured under DHA and EPA (25-100μM) in culture medium for 24h, 48h, 72h and 96h respectively. After treatment cell growth was then assessed and the experiment repeats three times.

### Apoptosis assay

The apoptosis of cells with different treatment were analyzed using a FITC Annexin V Apoptosis Detection Kit I (BD Biosciences, San Jose, CA) according to the manufacturer's instruction. Briefly, CRC cells were treated with DHA (75μM) or EPA (75μM) for 24h or 48h and were scraped and washed twice with ice-cold PBS and then re-suspended in 1×Binding Buffer at a concentration of 1×10^6^ cells/ml. Next 100μl of the solution, 5μl of FITC Annexin V and 5μl PI were sequentially transferred to a 5 ml culture tube followed by gently vortex and incubating for 15 min at 25°C in the dark. Finally 400 μl of 1×Binding Buffer was added to each tube and analyzed by flow cytometry within 1h.

### Western blot analysis

Cells were harvested followed by lysis and fixed amount of protein was loaded on a polyacrylamide gel followed by transfer to a PVDF membrane, followed by incubation with shaking overnight at 4°C with antibodies against phospho-YAP (Ser-127), total YAP/TAZ, phospho-MST1/2, total MST1, total MST2, phospho-LATS1(Ser-909), total LATS1, total LATS2 (all from Cell Signaling, Beverly, MA), GPR40, GPR120 (Abcam, Cambridge, United Kingdom), GAPDH and YAP (Santa Cruz Biotechnologies, Santa Cruz, CA) diluted in TBS containing 5% milk and 0.1% Tween-20. Signal was detected using the chemiluminescence (ECL) system (Millipore, Darmstadt, Germany).

### qRT-PCR

Total RNA was isolated with Trizol reagent (TaKaRa, Dalian, China) and all RNA was digested with DNase I (Takara, Dalian, China). The first-strand cDNA was synthesized using AMV Reverse Transcriptase (Thermo Fisher Scientific, Basingstoke, UK) according to the manufacturer's instructions. Real-time PCR was performed in triplicate with SYBR Green master mix (TaKaRa, Dalian, China) in the LightCycler® 96 Real-Time PCR System (Roche, Foster, CA). The experiment repeats three times. The sequences of primers for real-time PCR are listed in Supplementary Table 2.

### Confocal microscopy

HT-29 cells and LOVO cells were distributed per well in a 24-well plate and stimulated for 4 hours with DHA (75μM) or EPA (75μM). Cultured cells were washed with PBS and fixed with paraformaldehyde. After permeabilization with 1% Triton X-100 in PBS for 30 minutes at room temperature, cells were blocked in 5% (w/v) BSA in PBS and incubated with primary antibody YAP/TAZ (1:200; Cell Signaling, Beverly, MA) at 4°C for 16 hours. After incubating with primary antibody and washed with PBS for 5 minutes three times, cells were incubated with FITC-conjugated secondary antibodies (1:100) in PBS away from light for 1 h at room temperature. And the nuclei were stained with DAPI (5μg/ml). Cells were analyzed using confocal laser scanning microscope (LSM 700).

### Histology and immunohistochemistry

The specimen was sequentially fixed in 10% formalin for two days, transferred to ethanol of different concentration and embedded in paraffin in preparation for histopathological analysis. Thin sections (5 μm) were stained with hematoxylin-eosin (H&E) for histopathological study and subjected to immunohistochemical staining of target proteins with the avidin-biotin-peroxidase method. Slides were washed with PBS for five minutes three times and all the experimental steps were taken according to UNIV IHC detection kit operation instruction. Samples were incubated overnight with primary antibody GPR120 (Abcam, Cambridge, United Kingdom) (1:200), GPR40 (GeneTex, Nottingham, United Kingdom) (1:200), YAP/TAZ (Cell Signaling Inc Beverly, MA) (1:200), p-YAP (Cell Signaling, Beverly, MA) (1:200) at 4°C, followed by incubation with HRP-conjugated secondary IgG for 30 min at RT. After washing with PBS three times, 3-3′ diaminobenzidine (DAB) substrate chromogen solution (Envision Plus Kit, Dako Corp) was applied. The reaction was monitored by microscopy and was terminated when properly developed.

### Retroviral infection

Cells stably expressing empty vector, Myc-YAP (5SA) were generated by retroviral infection. 293 phoenix retrovirus packaging cells were transfected with empty vector, pQCXIH Myc-YAP (5SA) constructs (a kind gift from Dr. Bin Zhao of Zhejiang University, China) [48]. Forty-eight hours after transfection, retroviral supernatant was supplemented with 5 μg/ml polybrene, filtered through a 0.45 μm filter, and used to infect the indicated cells. Forty-eight hours after infection, cells were selected with either 200 μg/ml hygromycin in culture medium.

### Transfection of siRNA or plasmid DNA

Human YAP, LATS1, MST1, MST2, GPR40 and GPR120 specific and control random small interfering RNAs (siRNAs) were chemically synthesized by GenePharma Biological Technology (Shanghai, China). Dominant-negative Gαs mutant construct was purchased from Addgene (Cambride, MA, USA). Cells were transfected with the siRNAs using lipofectamine MAX according to the manufacturer's instructions (Invitrogen, Shanghai, China) and were analyzed by qRT-PCR and western blot to determine knockdown efficiency. Target sequences of these siRNA are listed in Supporting Table 3.

### Statistics

Statistical analysis was performed with the software package SPSS 13.0. All data were presented as means ± SEM for at least three independent experiments. The significant differences between two groups were evaluated by one-way ANOVA. Statistical significance was defined as *p*<0.05.

## SUPPLEMENTARY FIGURES AND TABLES



## Abbreviations

CRC: Colorectal cancer
ω-3 PUFAs: omega-3 polyunsaturated fatty acids
EPA: eicosapentaenoic acid
DHA: docosahexaenoic acid
MST1/2: mammalian sterile20-like kinases serine/threonine kinases 1/2
LATS1/2: large tumor suppressor serine/threonine protein kinases1/2
YAP: yes-associated protein
TAZ: transcriptional coactivators with PDZ-binding motif
SAV1: Salvador homologue 1
MOBs: Mps One Binder kinase activator proteins
GPRs: G-protein-coupled receptors
AOM/DSS: azoxymethane/dextran sulfate sodium
APC: adenomatous polyposis coli
PKA: cAMP-dependent protein kinase A
cAMP: cyclic adenosine monophosphate
AC: adenylate cyclase
