# Supplementary material for: G-protein-coupled receptors mediate ω-3 PUFAs-inhibited colorectal cancer by activating the Hippo pathway
Source: Oncotarget. 2016 Aug 5;7(36):58315–30. doi: 10.18632/oncotarget.11089 (PMC5295433; doi:10.18632/oncotarget.11089)
Supplement: Supplementary file 1 [file oncotarget-07-58315-s001.pdf]

G-protein-coupled receptors mediate ω-3 PUFAs-inhibited colorectal cancer by activating the Hippo pathway

SUPPLEMENTARY FIGURES AMD TABLES

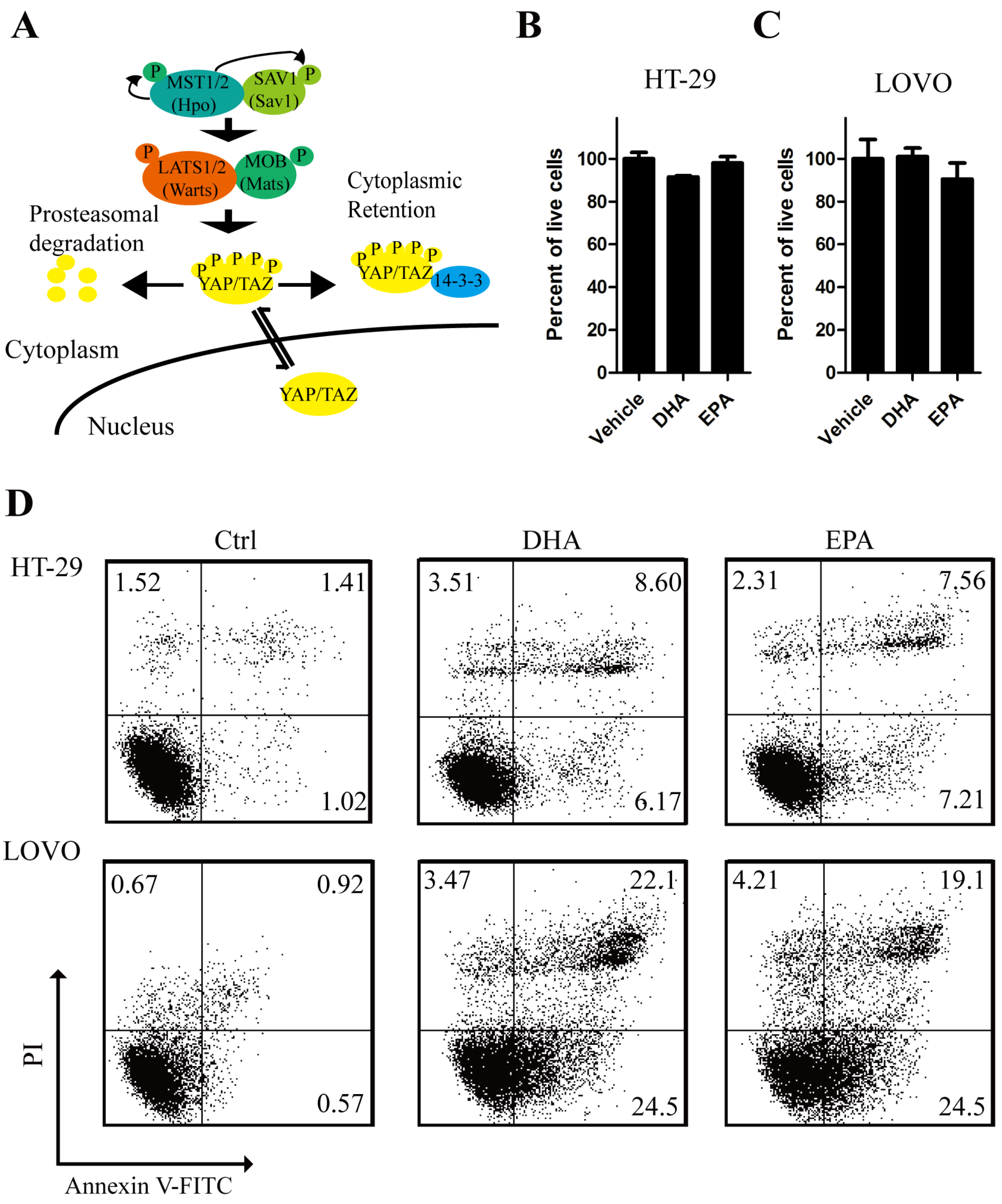

Supplementary Figure S1: DHA and EPA induce apoptosis of CRC cells. A. The schematic of the components of the Hippo pathway. **B** and **C.** HT-29 (B) and LOVO (C) cells growing in medium were treated with 100 µM either DHA or EPA, respectively, for 6 h and subsequently trypan blue staining was performed. The cell numbers were counted to measure the viability. Treatment with ethanol was used as a vehicle control. **D.** HT-29 and LOVO cells were treated with 75 µM DHA or EPA for 24 h, cell apoptosis was determined by FACS analysis. The data are expressed as the mean ± SEM for triplicate experiments. **P*<0.05.

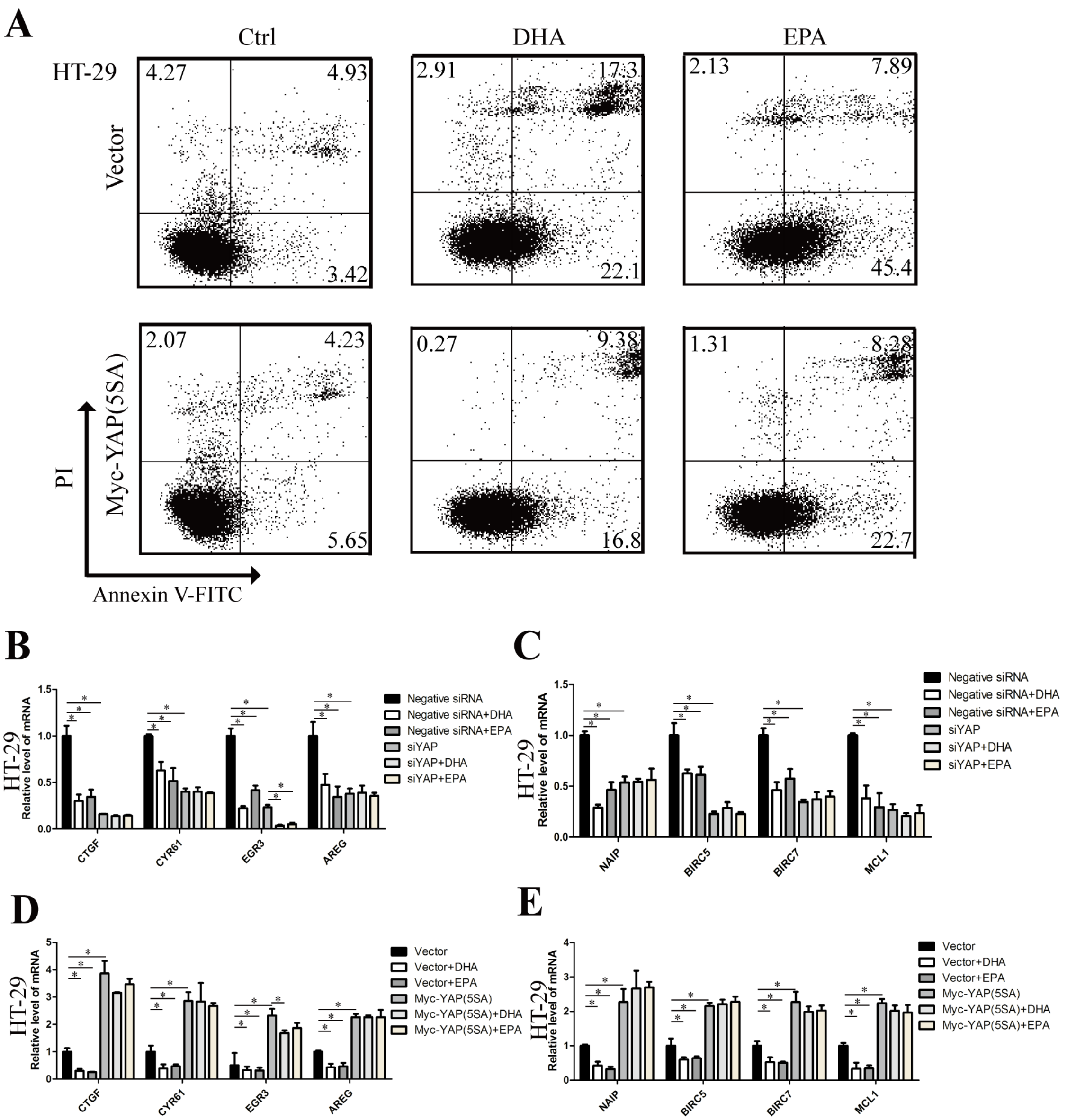

Supplementary Figure S2: ω-3 PUFAs inhibit proliferation and induce apoptosis of HT-29 cells via YAP. A. HT-29 cells were infected with empty vector or pQCXIH Myc-YAP (5SA) retroviral for 72 h. After infection, cells were treated with 75 µM DHA or EPA for additional 48 h, cell apoptosis was determined by FACS analysis. **B** and **C.** HT-29 cells were transfected with YAP siRNA for 48 h. After transfection, cells were treated with 75 µM DHA or EPA for additional 24 h. Total RNA was extracted and used for qRT-PCR analysis of the representative panel of pro-proliferative genes and anti-apoptosis genes. **D** and **E.** HT-29 cells were infected with empty vector or pQCXIH Myc-YAP (5SA) retroviral for 72 h. After infection, cells were treated with 75 µM DHA or EPA for additional 24 h. Total RNA was extracted and used for qRT-PCR analysis of the representative panel of pro-proliferative genes and anti-apoptosis genes. The data are expressed as the mean ± SEM for triplicate experiments. *$P<0.05$.

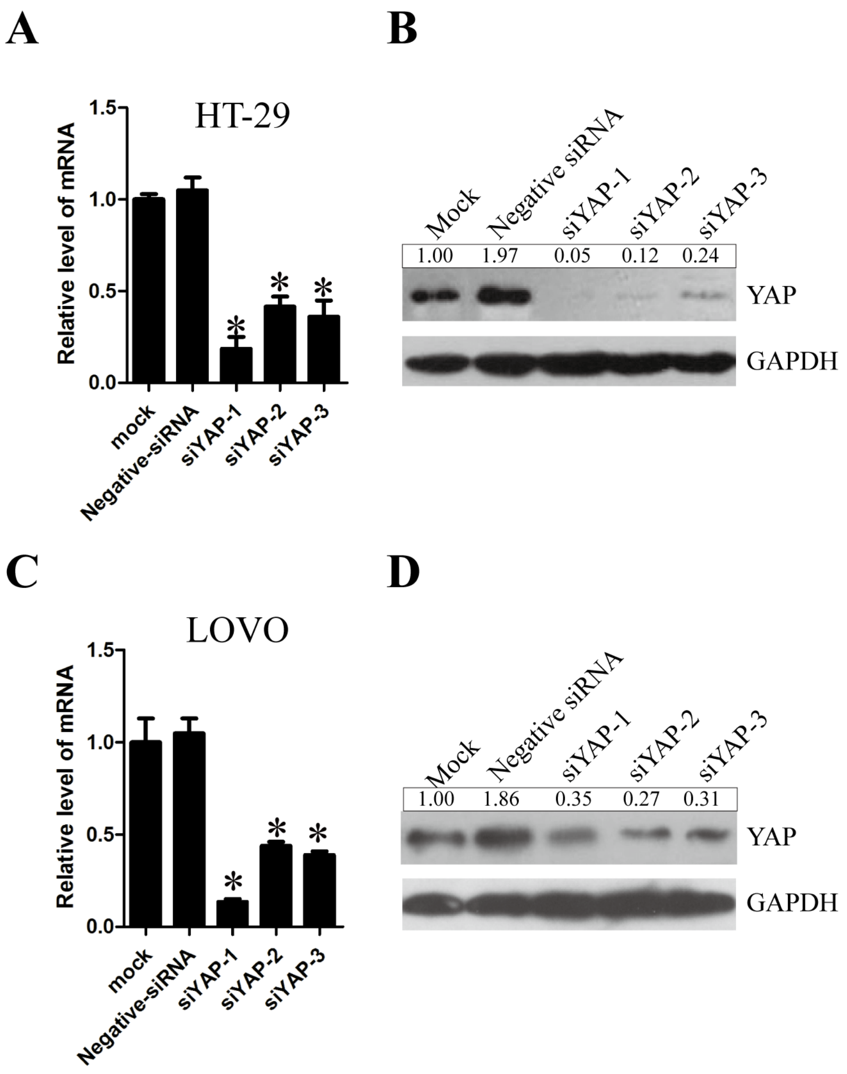

Supplementary Figure S3: The knockdown efficiency of YAP in CRC cells. A-D. The YAP knockdown was achieved by the transfection of the cells with YAP-targeting siRNAs sequence. The HT-29 (A and B) and LOVO (C and D) cells were either transfected with the negative control or the YAP-targeting siRNAs for 48 h and then the mRNA and protein were prepared for qRT-PCR analysis and western blot. The data are expressed as the mean ± SEM for triplicate experiments. *P<0.05.

Supplementary Table S1: Serum and colon mucosa fatty acid composition in control and ω-3 PUFAs group mice (weight % of total fatty acids)

PUFA	Control	ω-3 PUFAs
Serum		
LNA (18:2 n-6)	15.63±0.56	3.48±0.49**
AA (20:4 n-6)	11.34±0.84	8.76±2.03*
DTA (22:4 n-6)	0.06±0.03	0.17±0.03**
EPA (20:5 n-3)	0.16±0.05	6.24±1.27**
DPA (22:5 n-3)	0.23±0.07	0.37±0.18*
DHA (22:6 n-3)	4.53±1.72	13.34±3.26**
Colon mucosa		
LNA (18:2 n-6)	13.82±1.13	1.21±0.19**
AA (20:4 n-6)	7.36±1.35	4.48±1.17*
DTA (22:4 n-6)	1.07±0.22	0.64±0.17*
EPA (20:5 n-3)	0.43±0.16	5.47±1.63**
DPA (22:5 n-3)	0.46±0.08	0.79±0.31*
DHA (22:6 n-3)	1.53±0.43	12.35±1.93**

Values are means ± SEM, n=5 mice per group. */**indicate statistically significant differences with $P<0.05$ and $P<0.01$, respectively, compared to the control group.

Supplementary Table S2: The sequences of primers for real-time PCR

	antisense	sense
GAPDH (human)	GGCATGGACTGTGGTCATGAG	TGCACCACCAACTGCTTAGC
YAP (human)	CCAGGAATGGCTTCAAGGTA	CTCGAACCCCAGATGACTTC
GPR120 (human)	CTGTGCAGGAATGAGTGGAAG	CTGATGGAGGGTACTGGAAATG
GPR40 (human)	GTCTGGTCTTTGGGTTGGAG	GCAGGAGAGAGAGGCTGAAG
CTGF (human)	CTTGTGGCAAGTGAATTTCC	TGCTTTGAACGATCAGACAA
Cyr61(human)	AGCCTCGCATCCTATACAACC	TTCTTTCACAAGGCGGCACTC
AREG (human)	GTCATAGCCATAAATGATGAGTCG	AAATACTTTTTACCTTCGTGCACC
EGR3 (human)	GCAGCGACCACCTCACCAC	CCGCCTTCTTCTCCTTTTGCT
NAIP (human)	ACGAGACTCCCCATAGAAGAC	CTTCACCCTTATGTCGTACTTGG
BIRC5 (human)	AACAGCCGAGATGACCTCC	AACTTCAGGTGGATGAGGAGAC
BIRC7 (human)	GGCTCTGAGGAGTTGCGTC	AGGCCCCCATAGCAGAAGA
MCL1 (human)	AGACGATGTGAAATCG	TAACTAGCCAGTCCCG
β-actin (mouse)	ATGCCACAGGATTCCATACCCAA	CTCTAGACTTCGAGCAGGAGATGG
GAPDH (mouse)	TCAACAGCAACTCCCACTCTTCCA	TTGTCATTGAGAGCAATGCCAGCC
CTGF (mouse)	TCAACCTCAGACACTGGTTTCG	TAGAGCAGGTCTGTCTGCAAGC
Cyr61 (mouse)	AATACCGGCCCAAATACTGC	ATCTCTCCATCTTCGCATCG
AREG (mouse)	AGTGCTGTTGCTGCTGGTC	TCGCTTATGGTGGAAACCTC
EGR3 (mouse)	CCACCTCACCACTCACATCC	CTTGAGGTGGATCTTGGCGT
NAIP (mouse)	GGAGCCTGACTGAACTGAAGAA	GCAGAGAGCTCAGGGAGAAAT
BIRC5 (mouse)	ATAGAAAGCACTCCCCTGGC	TTGAAGCACCCTTCCTGAGC
BIRC7 (mouse)	AGATGTCCAGCCACCTCTAGT	AGAGTCCCTCAAGGCAAGTC
MCL1 (mouse)	GACTTGAAGCTGCCCAGGATT	TGGCCTCTCAGTGTTTGCTG

Supplementary Table S3: The sequences of siRNAs

Name	Forward 5' - 3'	Reverse 5' - 3'
siYAP-1#	GACGACCAAUAGCUCAGAUTT	AUCUGAGCUAUUGGUCGUCTT
siYAP-2#	GGUGAUACUAUCAACCAAATT	UUUGGUUGAUAGUAUCACCTT
siYAP-3#	CUGCCACCAAGCUAGAUAATT	UUAUCUAGCUUGGUGGCAGTT
siLATS1-1#	GGUAGUUCGUCUAUAUUAUTT	AUAAUAUAGACGAACYACCTT
siLATS1-2#	GAGCUGGAAAGGUUCUAAATT	UUUAGAACCUUUCCAGCUCTT
siLATS1-3#	GGUUGGGACUCCCAAUUAUTT	AUAAUUGGGAGUCCCAACCTT
siMST1-1#	GAGCUAUGGUCAGAUAACUTT	AGUUAUCUGACCAUAGCUCTT
siMST1-2#	GACAGAUGGAGCCAAUACUTT	AGUAUUGGCUCCAUCAGUCTT
SiMST2-1#	GCCCAUAUGUUGUAAAGUATT	UACUUUACAACAUAUGGGCTT
SiMST2-2#	GCUGGUCAGUUAACAGAUATT	UAUCUGUUAACUGACCAGCTT
SiMST2-3#	CCCACAAAUCCACCACCAATT	UUGGUGGUGGAUUUGUGGGTT
SiGPR40-1#	CCACUUCUUCCCACUCUAUTT	AUAGAGUGGGAAGAAGUGGTT
siGPR40-2#	UUGACCGGUGUGUUGAUGCTT	GCAUCAACACACCGGUCAATT
siGPR40-3#	CCUGGAGUGUGGUGCUUAATT	UUAAGCACCACACUCCAGGTT
SiGPR120-1#	GCCUUCACAUUUGCUAAUUTT	AAUUAGCAAAUGUGAAGGCTT
SiGPR120-2#	AAGAGGUUGAGUACCAGGCTT	GCCUGGUACUCAACCUCUUTT
SiGPR120-3#	AUUAGCAAAUGUGAAGGCCTT	GGCCUUCACAUUUGCUAAUTT
Negative siRNA	UUCUCCGAACGUGUCACGUTT	ACGUGACACGUUCGGAGAATT
